# Increased Insomnia Symptoms Predict the Onset of Back Pain among Employed Adults

**DOI:** 10.1371/journal.pone.0103591

**Published:** 2014-08-01

**Authors:** Maayan Agmon, Galit Armon

**Affiliations:** 1 School of Nursing, University of Haifa, Haifa, Israel; 2 Department of Psychology, University of Haifa, Haifa, Israel; University of Washington, United States of America

## Abstract

**Background:**

Back pain is among the most prevalent pain disorders causing chronic disability among adults, and insomnia is a common co-morbidity. However, whether insomnia precedes back pain or vice versa remains unclear. The current study tested the temporal association between insomnia and back pain.

**Methods:**

A longitudinal design was used to investigate whether changes in insomnia over time predict the onset of back pain and vice versa. The study was conducted on a cohort of active healthy working adults (N = 2,131, 34% women) at three time points (T1, T2, and T3) over a period of 3.7 years (range = 2.2–5.12) years. Logistic regression analysis was used to test whether increased insomnia symptoms from T1 to T2 predicted the onset of new back pain. Ordinary least squares regression was used to test whether the existence of back pain at T2 predicted an increase in insomnia from T2 to T3.

**Results:**

The results indicated that after controlling for socioeconomic variables, self-reported health, lifestyle behaviors, and anthropometrics, a T1–T2 increase in insomnia symptoms was associated with a 1.40-fold increased risk of back pain at T3 (OR = 1.40; 95% CI = 1.10–1.71). No support was found for reverse causation; i.e., that back pain predicts subsequent increase in insomnia.

**Conclusions:**

Insomnia appears to be a risk factor in the development of back pain in healthy individuals. However, no evidence of reverse causation was found.

## Introduction

An estimated 60% to 80% of the adult population experience back pain at some point in their lifetime [1]. In the workplace, back pain represents the single most costly condition in terms of its contribution to total workers’ compensation costs [2]. For example, in Europe, it accounts for 0.5–2% of the gross domestic product [3]. Most reviews on risk factors for back pain have concluded that its etiology is multifaceted, although approximately 90% of individuals suffering back pain have no identifiable cause whatsoever [4]. Thus, better identification of the risk factors and their relative contribution to the onset of back pain has the potential of advancing preventive health practices as well as reducing treatment costs. One of the most common comorbidities associated with back pain is insomnia and more than 50% of individuals suffering from back pain also report insomnia [5]. However, the nature of the association between these two conditions has yet to be clarified.

Insomnia is defined as difficulty initiating and/or maintaining sleep, prolonged awakening during the night, or waking up too early in the morning for more than a one-month period [6]. Compelling evidence, based on both self-reports and polysomnographic recordings, indicates an association between insomnia and back-pain [7], [8]. Furthermore, insomnia has been related to other types of pain, such as musculoskeletal [9], fibromyalgia [10], and arthritis [11]. Although the association between pain and insomnia is strong, the evidence showing causality is scarce.

Previous studies focusing on the back pain-insomnia association have suffered from critical methodological shortcomings: (i) cross-sectional studies with a limited ability to draw conclusions on causal links; (ii) small sample sizes undermining the generalizability of the findings; and (iii) inadequate adjustment for potential confounders, such as stress, depression, co-morbid chronic disease, obesity, and use of pain medication [7], [12].

In order to better understand the relationship between back pain and insomnia, this study evaluated the association between these two conditions among a cohort of healthy, working adults over three periods. The longitudinal design and the large, heterogeneous sample enabled us to examine, for the first time, the direction of the relationship between insomnia and back pain, after adjusting for socioeconomic factors, subjective reports of health status, lifestyle behaviors, and anthropometrics.

### Summary

#### What’s already known about this topic?

There is compelling evidence indicating an association between insomnia and back pain. While the association between pain and insomnia is strong, the evidence showing causality is scarce.

#### What does this study add?

This study indicates a unidirectional effect of insomnia on the incidence of back pain, based on an eight-year, longitudinal study among a cohort with a large sample of self-reported healthy working adults.

## Methods

### Study design

This longitudinal study was conducted through the Tel Aviv Medical Center Inflammation Survey (TAMCIS) between January 2003 and December 2011.

### Procedure

The study protocol was approved by the ethics committee of the Tel Aviv Sourasky Medical Center. Confidentiality was assured, and each participant signed a written informed consent document. In addition to the periodic health examinations, participants provided a medical history and underwent blood sampling after an overnight fast, a physical examination by a physician, urinalysis, stress ECG, spirometry, and vision and hearing function tests. The results of these examinations and the responses to the study questionnaire were recorded and entered into a computerized database.

### Back pain diagnosis

Diagnosis of back pain was assessed using two criteria: 1) medical record confirmation of a primary care physician visit at least once during the last 12 months for this reason and 2) the medical interview conducted by the physician at the medical center confirmed the persistence of the back pain. Both confirmed that the back pain persisted at least three months. A medical interview has been shown to have better diagnostic value than responses on self-administered questionnaires [13]. We coded respondents who reported having back pain at T1, T2, and T3 as = 1, otherwise = 0.

### Insomnia diagnosis

Insomnia was assessed by the first five questions of the Athens Insomnia Scale (AIS-5) [14]:

1. assessing difficulty with sleep induction
2. awakenings during the night
3. early morning awakening
4. sufficient total sleep time
5. overall quality of sleep

Responses ranged from 1 (never) to 7 (always). The AIS-5 has been validated on a clinical sample of insomniacs [14] and on an international non-clinical sample [15].

### Potential confounders and effect modifiers

The following variables associated with either back pain or insomnia were assessed: 1) demographic measures, including age, gender, and years of education; 2) lifestyle behaviors, including self-reported hours of strenuous leisure time physical activity per week [16], self-rated health [17], and smoking status (current or not); and 3) anthropometrics, including weight and height, as measured by a trained nurse and calculation of body mass index (BMI; kg/m^2^).

High-sensitivity C-reactive protein (hs-CRP) concentrations in serum were determined with the BN II Nephelometer g12 analyzer (DADE Behring, Marburg, Germany), as described by Rifai and colleagues [18]. This assay is based on particle-enhanced immunonephelometry and enables the measurement of extremely low hs-CRP concentrations (0.15 to 1000 mg/L).

Demographic measures and lifestyle behaviors were measured at T1, whereas self-rated health and anthropometrics (BMI and hs-CRP) were measured at T2 in order to test mediation effects. Finally, the number of days between T1 and T2, and between T2 and T3, referred to as time of follow-up, were documented.

### Statistical analysis

Considering an exploratory strategy, our primary goal was to evaluate the directionality of the relationship between back pain and insomnia. To determine whether increased insomnia levels predicted a subsequent onset of back pain, we first excluded from the analysis participants who reported having back pain at T1 or T2. We used logistic regression (SPSS 19 software) to predict the occurrence of back pain at T3 by T2 insomnia levels (being more proximal to the criterion relative to T1 insomnia). We then entered T1 insomnia as a control. The aforementioned control variables (age, gender, education level, physical activity intensity, self-rated health, smoking status, BMI, CRP, and time of follow-up) were subsequently entered.

To examine whether the occurrence of back pain predicted a subsequent increase in insomnia levels, we used an ordinary least squares (OLS) regression (SPSS 19 software). Because the predictor of interest was the dichotomy of reporting back pain at T2, but not at T1, we first excluded those participants who reported having back pain at T1. We predicted T3 insomnia first by T2 insomnia, thereby in effect predicting the change from T2 to T3 in insomnia [19]. We next entered the dichotomous variable of back pain at T2 and then controlled for the aforementioned possible confounders.

## Results

### Participants

**Figure 1:**
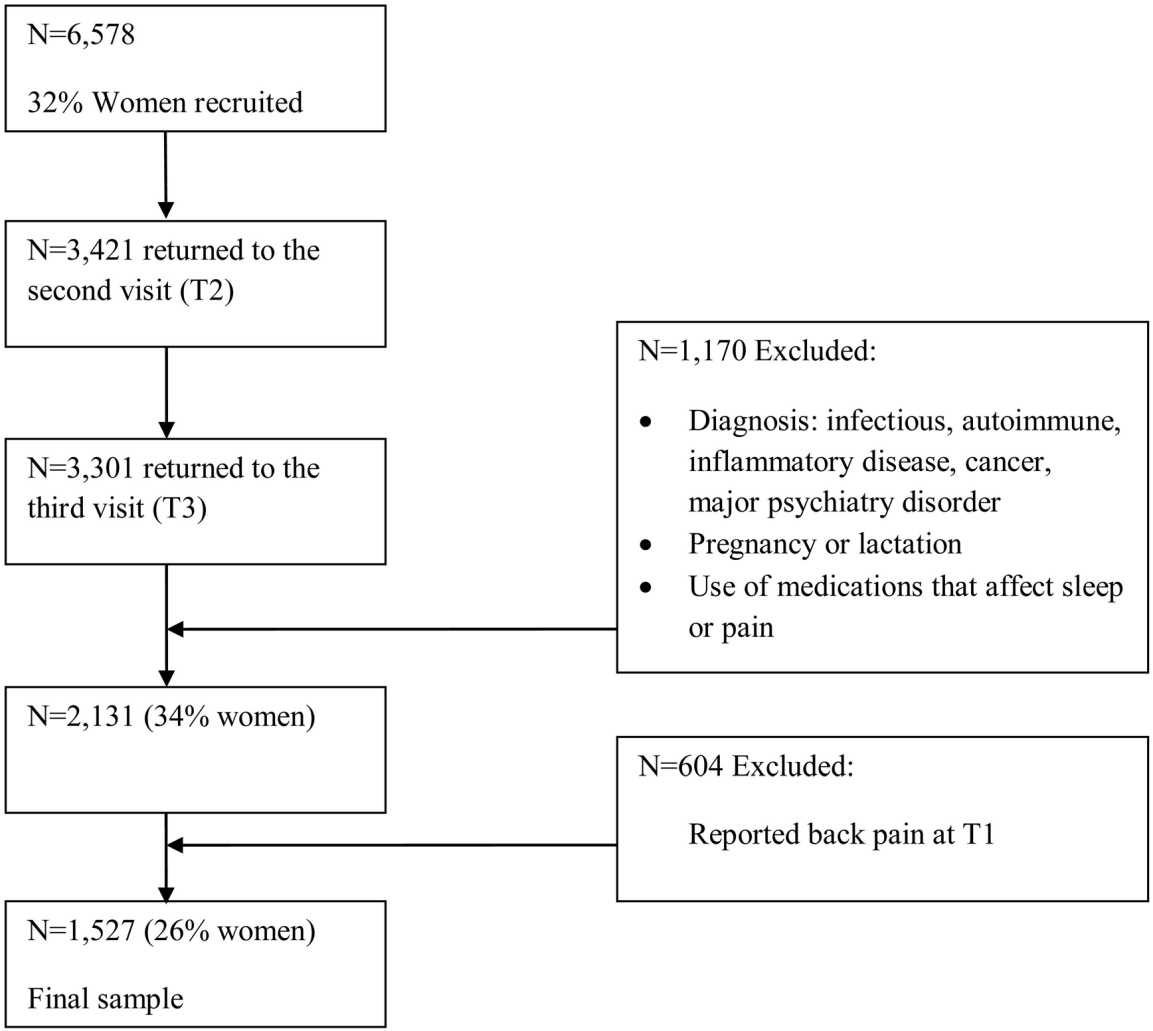
Participants Flow: Back pain predicts insomnia.

**Figure 2:**
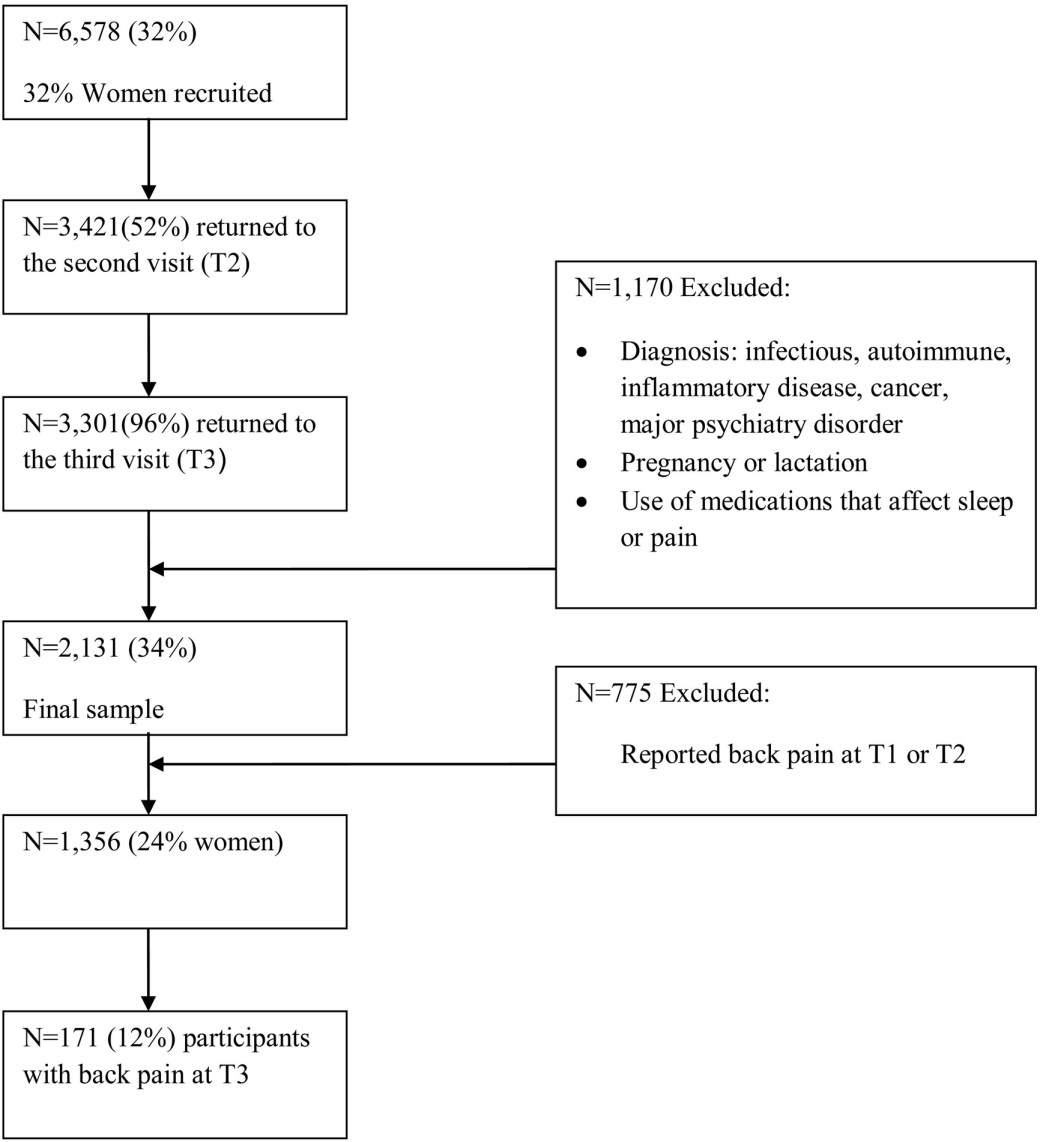
Participants Flow: Insomnia predicts back pain.

The study population (N = 7,150, 32% women), as shown in Figures 1 and 2, was comprised of employees attending the Center for Periodic Health Examinations at the Tel Aviv Sourasky Medical Center for a routine health examination at Time 1 (T1), Time 2 (T2), and Time 3 (T3). The average time lag between T1 and T2 was 18 months (range 11–19) and between T2 and T3 was 17 months (range 11–29). These periodic health examinations were provided to the study participants by their employer as a subsidized fringe benefit. Among this population, 92% (N = 6,578, 32% women) voluntarily agreed to participate in the TAMCIS study. Of those, 3,421 (52%) employees returned for a visit at T2, of which 3,301 (96%) returned for the third visit (T3). Study participants examined at T1 who did not return for follow-up examinations at T2 or T3 did not differ in socio-demographic or medical variables from those who completed all three examinations.

Of the participants who underwent all three examinations, 1,170 (35%) were excluded based on the following criteria: diagnosed infectious, autoimmune, or inflammatory disease; malignant/terminal disease (e.g., cancer); major psychiatric disorder (e.g., depression); pregnancy or lactation; and use of medications known to affect sleep or pain. Thus, the final sample consisted of 2,131 apparently healthy employees (34% women). Participants were a mean age of 46.20 (SD = 8.36) years of age at T1, and had a mean of 15.8 (SD = 2.66) years of education. They worked an average of 9.6 (SD = 2.25) hours per day.

### Insomnia predicted back pain

The data were examined to detect outliers or non-normal distributions (i.e. skewness >2.0 and kurtosis >5.0), but none were detected. To study whether insomnia predicted back pain, 775 respondents who reported having back pain at T1 or T2 were excluded from the analysis. Thus, the first analysis was conducted on 1,356 subjects (30% women, average age = 45.45; sd = 8.50) (see Table 1). A total of 171 (12%) subjects reported back pain at T3.

**Table 1:** Profile of participants without back pain at T1 and at T1 & T2.

Measure	Participants without back pain atT1 (N = 1,527)	Participants without back pain at T1 & T2 (N = 1,356)
Back pain at T3	11%	8%
Insomnia, T1, *Mean* *(SD)*	2.37 (.88)	2.34 (.87)
Insomnia, T2, *Mean* *(SD)*	2.34 (.89)	2.31 (.87)
Insomnia, T3, *Mean* *(SD)*	2.43 (.98)	2.40 (.96)
Gender *(1 = women), %*	26%	24%
Age (*year*s), *Mean (SD)*	45.55 (8.53)	45.45 (8.50)
Education *(graduated = 1), %*	15.92 (2.69)	15.95 (2.64)
Smoking (1 = Yes), T1, *%*	49%	39%
Physical activity, *Mean (SD)*	2.28 (1.98)	2.29 (1.96)
CRP, T2, *Mean* *(SD)*	2.44 (3.72)	2.44 (3.73)
BMI, T2, *Mean* *(SD)*	26.34 (3.86)	26.36 (3.87)
Self-rated health, *T2, Mean (SD)*	4.25 (.58)	4.26 (.58)

SD = standard deviation.

**Figure 3:**
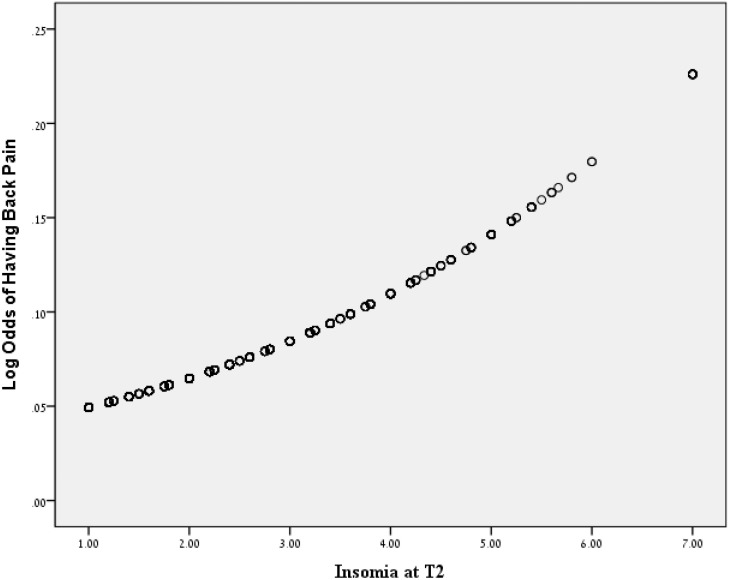
The relationship between insomnia at T2 (after controlling for insomnia at T1) and the log odds of having back pain.

Table 2 presents the results of the logistic regression analysis testing the hypothesis that an increase in insomnia symptoms at baseline would predict the presence of back pain at T3. After the aforementioned covariates were controlled, the change in insomnia levels from T1 to T2 was found to be associated with an increased risk of back pain from T2 to T3 (OR = 1.40; 95% CI = 1.10–1.71) (Figure 3). Although we used the change in insomnia symptoms from T1 to T2, both the T2 level alone and the level after controlling for T1 insomnia were significant predictors of the criterion. These results provide support for a unidirectional relationship between insomnia and back pain.

**Table 2:** Logistic regression analysis predicting the incidence of back pain at T3 relative to T2.

Measure	OR	95% CI
Insomnia, T1	.942	.72–1.24
Insomnia, T2	1.36*	1.27–1.51
Gender, T1	1.50*	1.05–2.13
Age, T1	1.01	.99–1.03
Education, T1	.99	.93–1.05
Smoking, T1	.97	.71–1.33
Physical activity, T1	1.06	.98–1.15
Self-rated health, T2	.87	.64–1.16
CRP, T2	.973	.92–1.03
BMI, T2	1.01	.96–1.05

N = 1,714.

*P<0.05.

Data are given as odds ratio, 95% confidence interval.

### Back pain did not predict insomnia

To study whether the occurrence of back pain predicted an increase in insomnia, we excluded from the analysis 604 respondents who reported having back pain at T1. Thus, our second analysis was conducted on 1,527 subjects (26% women, average age = 45.55; sd = 8.53).

The reverse-causation relationship – that the incidence of back pain at T2 predicted an increase in insomnia severity from T2 to T3 – was tested by OLS regressions, again controlling for the covariates described above. We found that the effect of the incidence of back pain at T2 was not a significant predictor (β = .02, ns) of insomnia at T3, controlling for T2 insomnia. (A table describing the results obtained in this regression is available from the first author upon request).

## Discussion

### Summary of findings and implications

Healthy working adults were almost one-and-a-half times more likely to experience back pain following insomnia symptoms or increased severity of insomnia over time, even after controlling for several potential confounding variables, such as socioeconomic status, self-rated health, lifestyle behaviors, and anthropometrics. However, the possibility of reverse causation, that back pain predicts an increase in insomnia symptoms, was not supported.

Thus, this study found a unidirectional effect of insomnia on the incidence of back pain, using data from an eight-year, longitudinal cohort study with a large sample of self-reported mentally and medically healthy working adults. These results extend the findings of other studies that indicated a cross-sectional association between insomnia and back pain [20]–[23]. Demographic variables, as well as variables that correlated with back pain and/or insomnia were also examined. Among these, female gender was a significant predictor of back pain. Indeed, back pain is more common among women than men [24].

Thus, the findings of the present study suggest that insomnia serves as a marker for individuals at increased risk for back pain. Health practitioners taking a medical history and conducting physical examinations should inquire about primary insomnia, particularly in cases of patient reports of non-specific back pain. These findings suggest that insomnia may lead to back pain, contrary to the assumption that back pain precedes insomnia. As such, the findings highlight the need to treat both conditions and prescribe combined treatment, while taking a preventive approach to non-specific back pain in order to promote sleep quality.

### Possible biological mechanisms

Several mechanisms may explain the association between insomnia and back pain. One is that both insomnia and non-specific back pain are caused by a third element, which we have yet to identify. Insomnia might be more susceptible to this third element and so predispose to back pain. Another possible explanation is that insomnia indeed leads to non-specific back pain.

It is important to note that there is substantial overlap between variables supporting both of these explanations; consequently, it is unlikely that the cause is derived solely from one explanation or the other. In fact, a common dopaminergic abnormality was found to be associated with both insomnia and back pain [25], suggesting that this abnormality may act as a mediator in the insomnia-back pain link. Recent studies have discussed the potential effects of dopamine on pain threshold and pain intensity among patients with different pain-related conditions [26]. However, a clear understanding of this mechanism has yet to be achieved [27].

Stress may be another mediating mechanism underlying both disturbances. Individuals with insomnia perceive their lives as stressful and are likely to experience chronic tension and restlessness [28]. This, in turn, may increase muscle tension and decrease micro-pauses in muscle activity, which leads to back pain [29], [30]. In addition, stress is related to activation of the sympathetic nervous system. There is solid evidence that arousal of the sympathetic nervous system is evident in insomnia patients [31]. Such sympathetic nervous activity may lead to norepinephrine secretion, which in turn heightens muscle tone and increases the risk for pain and injuries of muscular origin. Indeed, Elfering et al. [30] found a positive association between norepinephrine levels and back pain. Moreover, continuous arousal of the sympathetic nervous system enhances the inflammatory process [32], [33], thereby increasing the concentration of cytokines and inflammatory mediators, which is associated with insufficient sleep [34], [35]. In the current study, we did not find support for this pathway given that hs-CRP, an inflammatory biomarker that has already been related to conditions associated with back pain [36]–[39] and insomnia [40], did not mediate the insomnia-back pain association. This may be attributed to the fact that high hs-CRP levels are more strongly correlated with non-restorative sleep, a subtype of insomnia symptoms [40], which was not measured here.

Another possible explanation supporting the assumption that insomnia precedes back pain is related to the key role of insomnia in enhancing pain sensitivity [41], [42]. Individuals with insomnia experience spontaneous pain more frequently and intensely, exhibit a higher sensitivity to evoked heat and pressure pain, and have dysfunctional pain inhibition processing compared to healthy individuals [43]. These findings might be explained by conditioned pain modulation (CPM) in the human psychophysical lab test for the ‘diffuse noxious inhibitory control’ (DNIC) phenomenon [44], exploring the ‘pain inhibits pain’ mechanism in patients, representing decreasing pain inhibition. Many recent publications have reported less efficient CPM in various chronic pain disorders [45]–[47]. These reports are in line with those of Haack and colleagues [43], who found increased spontaneous pain and pain sensitivity in patients with insomnia, which may lead to back pain. We suggest that future studies investigate these potential mediating mechanisms.

### Strengths and limitations

The study had several strengths. First, it was based on a large, heterogeneous sample. Second, subjects with chronic physical or mental illness who might confound the results were carefully excluded. Third, the longitudinal design using three waves of data allowed for testing reverse causation and the interpretation of causality in the link between increased insomnia severity over time and the development of back pain [48]. This has obvious advantages over testing cross-sectional relationships, as it controls for the confounding influence of time [49]. However, several limitations should be considered.

### Selection bias

The sample of workers recruited at a single site may not be representative of other employed adults. Most were highly educated white-collar workers who exhibited generally good health behavior patterns, smoked little, and exercised regularly. Due to these health habits, the participants may have been more resilient to the effects of insomnia on back pain. Moreover, employed individuals are often healthier than the general population due to the “Healthy Volunteer Effect” [50]. Nevertheless, it is likely that the finding would be more robust in a sample of working adults that included participants who were less educated and less healthy than those in the current study.

### Measurement limitations

This study did not include a general sleep questionnaire, which would have provided subjective estimates of important variables, such as time to fall asleep at bedtime, number of awakenings per night, time spent awake during the night, number of nights per week that end with a long early final awakening, and number of hours spent in bed per night. Such information could support the findings from the more qualitative information collected with the Brief Athens Insomnia Scale, which was validated in a clinical sample of insomniacs [14]. However, other studies have shown that subjective reports of insomnia correlate strongly with more objective measures, such as polysomnographic examinations [51].

## Conclusions

The finding that healthy employed individuals were almost one-and-a-half times more likely to experience back pain if they had insomnia symptoms, even after adjusting for important confounders has the potential to improve both the diagnosis and treatment of this population.
